# Rapid prediction of NMR spectral properties with quantified uncertainty

**DOI:** 10.1186/s13321-019-0374-3

**Published:** 2019-08-06

**Authors:** Eric Jonas, Stefan Kuhn

**Affiliations:** 1 Department of Computer Science, University of Chicago, Chicago, USA; 2 School of Computer Science and Informatics, Leicester, UK

**Keywords:** NMR, Machine learning, DFT

## Abstract

Accurate calculation of specific spectral properties for NMR is an important step for molecular structure elucidation. Here we report the development of a novel machine learning technique for accurately predicting chemical shifts of both ${^1\mathrm{H}}$   and ${^{13}\mathrm{C}}$ nuclei which exceeds DFT-accessible accuracy for ${^{13}\mathrm{C}}$ and ${^1\mathrm{H}}$ for a subset of nuclei, while being orders of magnitude more performant. Our method produces estimates of uncertainty, allowing for robust and confident predictions, and suggests future avenues for improved performance.

**Electronic supplementary material:**

The online version of this article (10.1186/s13321-019-0374-3) contains supplementary material, which is available to authorized users.

## Introduction

Nuclear magnetic resonance (NMR) spectroscopy is an established method in analytical chemistry. In contrast to other spectroscopic techniques like mass spectrometry (MS), it is non-destructive; in contrast to various optical spectroscopic techniques, it can often give sufficient information to completely elucidate the structure of an unknown molecule. Therefore NMR is an essential tool in many fields of chemistry and biology.

In NMR one major source of information is the specific resonance frequency, termed the chemical shift, at a given spin-active nucleus in a molecule (here we focus on ${^1\mathrm{H}}$ and ${^{13}\mathrm{C}}$). The local molecular environment around a nucleus determines its chemical shift, leading to various “rules of thumb” that are taught to undergraduate organic chemists. The development of “pure-shift” NMR pulse sequences [1], which can accurately measure chemical shift values with neither homo- nor heteronuclear coupling, makes it even easier to identify precise chemical shift values in crowded spectra.

The association of particular chemical shift values with certain molecular motifs becomes the knowledge of any working organic chemist, but early on efforts were made to computationally assess these properties. One of the earliest was HOSE codes [2], which attempt to featurize (summarize) the neighborhood around each atom in concentric spheres, and then use a nearest-neighbor approach to predict the particular shift value. Simultaneously, advances in calculation of NMR properties from first principles (ab initio) made considerable progress with the introduction of Density Functional Theory; today’s DFT-based methods can be quite accurate [3] and reasonably turnkey, if time-consuming, protocols have been developed for their application [4].

Recently deep neural networks have made incredible progress in various machine learning disciplines, including vision and audition. In chemistry, recently-developed graph neural networks [5] and their extensions [6] have led to efforts to predict whole-molecule properties [7] (such as energy of formation, logP, and others) based on large molecular datasets. In an effort to predict per-nucleus chemical shift values, we adopt convolutional graph networks to predict both per-atom properties and provide an estimate of their own uncertainty.

## Methods

A convolutional graphical neural network is a specific type of relational neural network [6] which attempts to learn a set of local filters (convolutions) on the graph that predict properties for a given node. At each layer in our vertex-focused convolutional graph network, we associate with each vertex $v_i$ a *D*-dimensional vector of features $l^{(n)}_i \in \mathbb {R}^D$. Each layer computes a new feature vector $l_i^{(n+1)}$ for vertex $v_i$ as a nonlinear function of the vertices it is connected to,

1 $$\begin{aligned} l_i^{(n+1)}[d] = f\left( \sum _{v_j \in \mathcal {N}(v_i)} (w_d^{(n)})^T l_j^{(n)}\right) \end{aligned}$$

In this case, *f* is a per-vertex nonlinear function, and the neighborhood calculations are performed based on specific bond orders. We concatenate multiple layers of this type, and train the per-layer weight matrices $W^{(n)}$ via stochastic gradient descent. This gives us a principled way of learning a set of local, connectivity-constrained nonlinear operations on each graph.

We stack each layer’s features into a *M* by *D* matrix $L^{(n)} \in \mathbb {R}^{M \times D}$ where *M* is the maximum number of possible vertices in a graph (in our case, 64). For a given molecule we create 4 adjacency matrices $G_1, G_{1.5}, G_2, G_3$ where entry $g_{i,j}$ indicates a bond of the relevant order between vertices $v_i$ and $v_j$. We then normalize each *G* matrix to $\widetilde{G} = (r^{-\frac{1}{2}})^T (G + I) (r^{-\frac{1}{2}})$ where *r* is the vector of row-sums of $G+I$. This is akin to Eq. 2 in [5]. Then we compute

2 $$\begin{aligned} \widetilde{L}^{(n+1)} = \phi \left(\psi (\widetilde{G}L^{(n)} W^{(n)} )\right) \end{aligned}$$

where $\psi$ is $\max$ over bond orders, and $\phi$ is a per-vertex rectified linear unit. Thus each layer consists of a $W^{(n)} \in \mathbb {R}^{D_{n+1} \times D_{n}}$ matrix of parameters that takes in a $L^{(n)} \in \mathbb {R}^{M \times D_{n}}$ matrix of per-vertex features and outputs a $L^{(n+1)} \in \mathbb {R}^{M \times D_{n+1}}$ matrix of features. We use a residual structure, such that $L^{(n+1)} = \widetilde{L}^{(n+1)} + L^{(n)}$.

In our case, all layers have the same number of input and output features except for the first. We use 10 layers of 2048 features, and the first layer takes per-atom input features which are derived from each atom and its environment, and include those in Table 1. Note some numerical features are encoded as binary vectors (so-called *one-hot* encoding) where the *i*th entry in the vector corresponds to a feature value of *i*. Thus the output of the graph portion of our network is a 2048-element feature per vertex.

**Table 1:** Per-atom input features computed from RDKit molecules

Feature	Description	Number
Atomic number		1
Atomic number	One-hot encoded {H, C, O, N, P, S, F, Cl}	8
Valence		1
Valence	One-hot encoded 1–6	6
Aromaticity	Does RDKit identify this atom as being part of an aromatic structure	1
Hybridization state	One-hot encoded *s*, *sp*, $sp^2$, $sp^3$, $sp^3d$, $sp^3d^2$	6
Formal charge	Does this atom have a net charge, one-hot encoded {$-\,1$, 0, $+\,1$}	3
Default valence	One-hot encoded 1–6	6
Rings	Is this atom the member of a ring , one-hot encoded 3–7	5
Total		37

This feature is fed through two 128-dimensional linear units to calculate the uncertainty value, and a stacked collection of 3 fully-connected residual blocks before a final linear layer as output (see Table 2). The network uses rectified nonlinearities (ReLUs) exclusively for nonlinear activations.

**Table 2:** Post-graph-network linear layers for predicting per-atom $\mu$ and $\sigma$

	$\mu$	$\sigma$
Input	2048	2048
	Linear/ReLU 2048$\rightarrow$128	Linear/ReLU 2048$\rightarrow$128
	Linear/ReLU 128$\rightarrow$128	Linear/ReLU 128 $\rightarrow$ 1
	128-residual	output $\sigma$
	128-residual	
	128-residual	
	Linear/ReLU 128$\rightarrow$128	
	output $\mu$	

We train separate networks for ${^{13}\mathrm{C}}$ and ${^1\mathrm{H}}$ chemical shifts. Each network takes approximately 4 hours to train on a V100 GPU on an Amazon Web Services p3.2xlarge instance. All code is implemented in PyTorch 0.4.1.

A challenge with applying machine learning techniques (as opposed to more conventional statistical or probabilistic techniques) to scientific data is these methods focus on producing point estimates. In a sense, a neural network doesn’t know what it doesn’t know. This can make their application challenging as for many applications minimization of an error metric is less useful without some understanding of how bad that error might be. Taking inspiration from recent attempts to resolve these challenges, we augment our network to produce an estimate both of the mean of a chemical shift value and the variance.

There has recently been tremendous research interest in getting deep learning methods to provide measures of uncertainty with their predictions, including injecting noise into the network at prediction time [8] and minimizing a variational objective [9]. We adopt a simpler approach of having our network simply calculate the mean and an uncertainty value for each predicted shift value. We then minimize the scaled mean squared error of this model, giving a loss function of

3 $$\begin{aligned} \frac{(y_i - \mu _i)^2}{2\sigma _i^2} + \sigma _i \end{aligned}$$

where $y_i$ is the predicted value, $\mu _i$ is the true value, and $\sigma _i$ is the uncertainty value. Inspection reveals this to be functionally similar to the negative log likelihood of a normal distribution with variance $\sigma _i^2$. We independently predict both $\mu$ and $\sigma$ for each vertex, and our loss function zeros out the loss at unobserved nuclei.

**Fig. 1:**
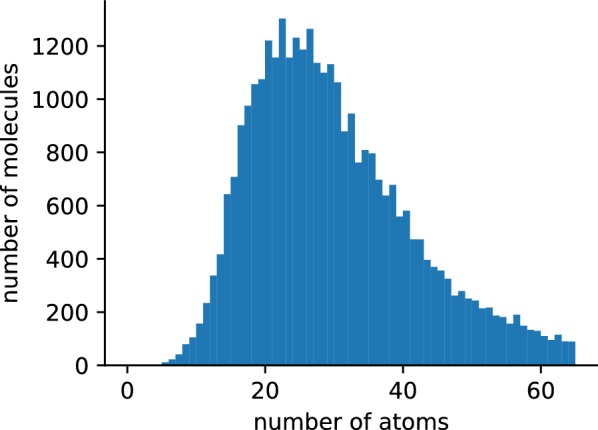
Distribution of atom sizes in our identified data subset

For input data we use the data available in nmrshiftdb2 [10], a user-contributed database of 43,468 molecules and 52,731 spectra.^1^ We identified all molecules in nmrshiftdb2 with annotated ${^{13}\mathrm{C}}$ or ${^1\mathrm{H}}$ chemical shift values containing only the elements {H, C, O, N, P, S, F, Cl}, excluding elements with a very low occurrence in nmrshiftdb2. We excluded any molecules which failed to successfully pass the sanitize process in RDKit [11] and had no more than 64 atoms. This left us with a total of 32,538 molecules with an average size of 29 atoms. Figure 1 shows the distribution of molecule sizes. 11,616 ${^{13}\mathrm{C}}$ nuclei had more than one measurement and 1013 ${^1\mathrm{H}}$ nuclei had more than one measurement; if we assume the “true” value for a nucleus is the average of all its values, and then compute the mean absolute error we arrive at 0.51 ppm for ${^{13}\mathrm{C}}$ and 0.09 ppm for ${^1\mathrm{H}}$, suggesting an intrinsic measurement variability on this order for this dataset.

Multiple molecules in nmrshiftdb2 have multiple spectra, so we made sure that all measurements were in a given train/test split. We trained on 80% of the data and held out 20% for evaluation. We validated this train/test split via SMILES strings, ensuring that no molecule in the train set had the same SMILES string as one in the test set. The dataset used was Revision 1624 from the nmrshiftdb2 on sourceforge.net. They can be accessed via https://sourceforge.net/p/nmrshiftdb2/code/1624/.

## Results

**Fig. 2:**
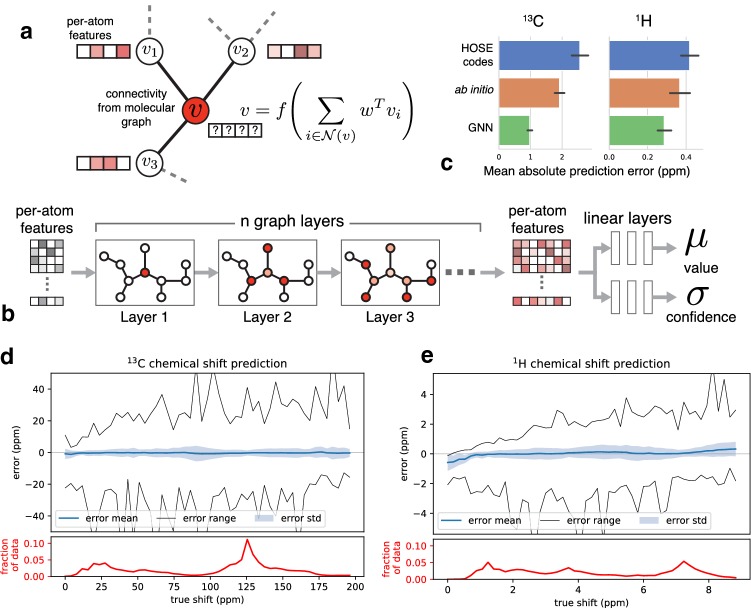
**a** A convolutional graph neural network computes per-vertex (atom) parameters by performing a weighted linear combination of neighboring vertices, and then passing the result through a nonlinearity. **b** Successive layers serve to aggregate information from more and more distant vertices (atoms), respecting the connectivity of the graph. The resulting per-vertex features are then passed through a series of linear layers to estimate a chemical shift value and a confidence level for each vertex (atom). **c** Mean absolute prediction error for ${^{13}\mathrm{C}}$ and ${^1\mathrm{H}}$ chemical shifts, comparing classical HOSE codes, ab initio calculations, and our graphical neural network. Error bars are bootstrap-estimated 95% confidence intervals for the mean. **d** GNN chemical shift errors for ${^{13}\mathrm{C}}$ as a function of true shift (ppm), showing mean error, standard deviation of the error, and max error. Lower panel shows the fraction of the training data present at that chemical shift. **e** Same as (**d**) but for ${^1\mathrm{H}}$

We show the accuracy of our method for both ${^{13}\mathrm{C}}$ and ${^1\mathrm{H}}$ chemical shift prediction as a function of the true ppm in Fig. 2d and e as well as the range of prediction errors, at a level corresponding to predicting 95% of the nuclei in the dataset. Our accuracy closely tracks the fraction of chemical shifts observed in the initial data, across the entire dataset.

**Fig. 3:**
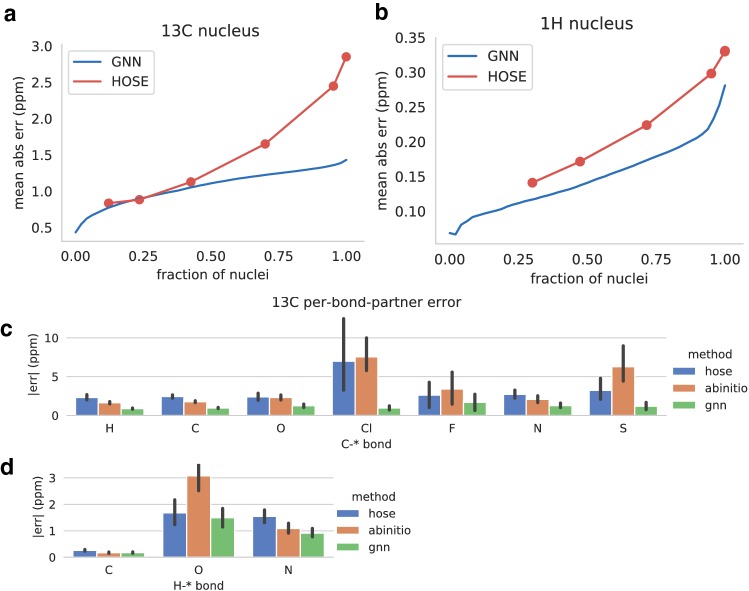
Method comparison. **a** Mean absolute error (ppm) for ${^{13}\mathrm{C}}$  nuclei predicted at a given confidence interval for our network and for HOSE codes. The x-axis is the fraction of nuclei we predict at that confidence interval. HOSE codes provide a discrete (sphere-number) measure of confidence. Comparison is across all selected NMRShiftDB molecules. **b** same as a. for ${^1\mathrm{H}}$. **c** Prediction errors broken down by bonding partner. Errors are worse for more electronegative bonding partners as expected. **d** Same as c for ${^1\mathrm{H}}$. Error bars are bootstrap-estimated 95% confidence intervals for the mean

Evaluation of uncertainty-preserving methods must take into account the fraction of data at a given uncertainty level when making predictions. Different methods will have differing levels of certainty for different fractions of the data. Depending on the use case, a method may be very confident (and accurate) on a small minority of the data, and substantially less confident (and accurate) on a larger portion of data. It can be challenging to compare this to a method which is uniformly confident and more accurate on average across all data. To resolve this, we compute the fraction of nuclei that the method would predict at a given confidence threshold, and then compute the mean error of that fraction. Figure 3a, b show the comparison between HOSE and GNNs for ${^{13}\mathrm{C}}$ and ${^1\mathrm{H}}$, respectively. We see that GNNs tie or outperform HOSE codes for all thresholds, but HOSE’s performance on ${^{13}\mathrm{C}}$  is very competitive in the high-confidence regime. This makes sense, as HOSE codes are fundamentally a nearest-neighbor method, and can perform exceptionally well when there are very-similar molecules in the training data (Table 3).

**Table 3:** Mean average error for predicted nuclei subset at various confidence thresholds

Frac data (%)	${^{13}\mathrm{C}}$ MAE		${^1\mathrm{H}}$ MAE	
	GNN	HOSE	GNN	HOSE
100	1.43	2.85	0.28	0.33
95	1.35	1.65	0.22	0.22
80	1.27	1.65	0.18	0.22
50	1.11	1.13	0.14	0.17
25	0.91	0.88	0.11	
10	0.71		0.09	

For example, at the level of confidence were both methods are able to predict 80% of the nuclei, the GNN has a MAE of 1.27 ppm and HOSE codes have a MAE of 1.65 ppm for ${^{13}\mathrm{C}}$

To compare with DFT methods we identified a subset of 177 molecules in nmrshiftdb which had the greatest number of independent spectral measurements, and followed best practices for ab initio calculation chemical shift values (see Additional file 1: Section 2.2). Table 4 shows the results for per-nucleus and per-molecule estimation for both ${^{13}\mathrm{C}}$ and ${^1\mathrm{H}}$ nuclei. DFT methods struggle to calculate chemical shifts for atoms bonded to halogens, as shown in Fig. 3c for ${^{13}\mathrm{C}}$ and Fig. 3d for ${^1\mathrm{H}}$.

**Table 4:** Comparison of chemical shift prediction accuracy for 177-molecule subset across methods, for various metrics

Nucleus	Method	mol MAE	mol RMSE	MAE
${^{13}\mathrm{C}}$	GNN	1.01	1.20	0.97
	HOSE	3.42	4.24	2.56
	Ab Initio	2.24	2.73	1.92
${^1\mathrm{H}}$	GNN	0.30	0.40	0.29
	HOSE	0.45	0.59	0.42
	Ab Initio	0.39	0.57	0.37

“mol“ methods are first averaged per-mol (see Additional file 1: Section 1). All predictions are made without uncertainty filtering for GNN and HOSE

We evaluated runtime performance in Table 5 on per-molecule and per-predicted-nucleus runtimes. While our method is faster than computing HOSE codes this may be due to implementation differences, with our neural network running on a modern GPU and HOSE code invocation requiring a database-lookup. Both methods are orders of magnitude faster than performing ab initio calculations.

**Table 5:** Runtime comparison

Method	Note	Per-mol	Per-nucleus
GNN^a^		3.6 ms	56 μs
HOSE^b^	${^{13}\mathrm{C}}$	29 ms	2 ms
	${^1\mathrm{H}}$	34 ms	4 ms
DFT^c^	geom. opt	556 s	36 s
	GIAO	256 s	16 s

^a^ GNN computes a fixed number of nuclei (64) at all times

^b^ HOSE predictions, by virtue of being a nearest-neighborhood method, take longer on larger training datasets

^c^ DFT comparison is the mean of wall-clock runtime, but this should be interpreted cautiously given the tremendous variance in number of conformers per molecule and the $O(n^3)$ scaling

**Fig. 4:**
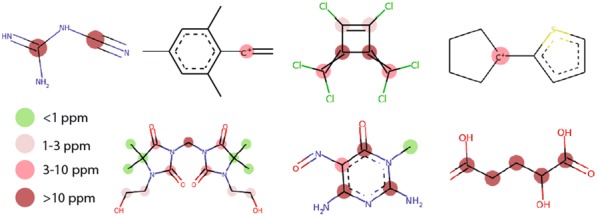
A subset of the molecules in our test set for which we had the highest RMSE error. Many have exotic structures, are radicals, or have an unusual abundance of halogens. Upon closer examination, the final example molecule has its predicted shifts off by a consistent 5 ppm, suggesting a possible reference error

Finally, we looked at some of the molecules in the test set where our method performed the worst. We can see in Fig. 4 that many have exotic structures, are radicals, or have an unusual abundance of halogens. Upon closer examination, the final example molecule has its predicted shifts off by a consistent 5 ppm, suggesting a possible reference error. As the data in nmrshiftdb2 is user-contributed, we were unable to confirm this is in fact the source of the error, but this highlights a potential unexpected use of our method in rapid checking of user-submitted structures and peaks.

## Discussion

While our approach shows the promise of graphical neural networks with uncertainty in predicting per-nucleus properties such as chemical shift, we note several caveats. First, our method ignores solvent and temperature effects, which are known to alter chemical shift values in experiments. The vast majority of user-contributed NMR spectra in NMRShiftDB are contributed without an indicated solvent or temperature, and this likely contributes significantly to noise in our training dataset. A promising avenue would be to combine high-throughput ab initio data with high-quality careful experimental data (transfer learning). Second, by focusing on purely connectivity (bond-order) information, we are ignoring stereochemical effects and geometry-specific effects. It may be the case that explicitly incorporating geometric properties into our model would improve prediction accuracy, but this is left for future work.

Finally, any comparison with ab initio techniques is going to be extremely sensitive to the level of theory and molecular dataset used for comparison. More accurate incorporation of conformational effects [12] can yield considerably more-accurate calculated shift values, at the expense of considerable computation time. Also, any comparisons of method performance are going to be sensitive to the molecules included in the validation set.

We view our method as a useful step towards the fully ML-based prediction of nuclear magnetic resonance spectra for structure elucidation. Our method is integrated with the NMRShiftDB database, and all code and data are available under a BSD license. Additional properties, such as indirect dipolar coupling (J-coupling) coefficients and nuclear Overhauser-effect (NOE) couplings should be amenable to the methods we describe in this paper. Combined with recent work in other spectroscopic techniques, such as solid-state NMR chemical shifts [13] and IR spectroscopy[14], we can imagine a regime where the prediction of multiple spectroscopic parameters could be automated and nearly instantaneous for many chemical tasks.

## Conclusion

We have demonstrated the use of deep neural networks for predicting single-atom properties of molecules, in particular NMR shifts. We achieved a precision of 1.2 ppm mol RMSE for ${^{13}\mathrm{C}}$  and 0.4 ppm mol RMSE for ${^1\mathrm{H}}$ shifts. This is significantly better than conventional HOSE code-based prediction, and can achieve the reliability of a DFT-based prediction for an identified subset of molecules. Finally, our method incorporates an uncertainty measurement for predictions, enabling confident predictions for subsequent structure elucidation tasks. We have integrated our approach with the online NMRShiftDB website, allowing users to upload candidate structures and receive rapid predictions for chemical shift values, and anticipate improving both accuracy and molecular coverage in the future.

## Additional files

**Additional file 1: Table S1.** List of active ingredients in Radix *Salviae Miltiorrhizae* (RSM).

**Additional file 2: Table S2.** List of the chemical compounds and putative targets of RSM following screening.

## Data Availability

The assigned NMR data in nmrshiftdb2 can be browsed at https://www.nmrshiftdb.org and are available from https://sourceforge.net/p/nmrshiftdb2/code/HEAD/tree/trunk/snapshots/ for download. The source code of the project along with data are available in the Additional file 2.

## Footnotes

1 As of 15 November 2018.
