# Supplementary material for: Rapid prediction of NMR spectral properties with quantified uncertainty
Source: J Cheminform. 2019 Aug 6;11:50. doi: 10.1186/s13321-019-0374-3 (PMC6684566; doi:10.1186/s13321-019-0374-3)
Supplement: Supplementary file 1 — Additional file 1: Table S1. List of active ingredients in Radix Salviae Miltiorrhizae (RSM). [file 13321_2019_374_MOESM1_ESM.pdf]

# 1 Metrics

Metrics for chemical shift evaluation differ across the literature. We have not found any metric to be better than any other, and carefully define each here to remove ambiguity. In particular, in the literature the difference between per-molecule MAE and overall (per-nucleus) MAE is often confused. Per-molecule methods are harder to consistently apply when the prediction method is estimating fewer than the total number of shifts per molecule (due to uncertainty), so we focus most on total MAE in this paper.

We adopt the following metrics, assuming our dataset has $M$ total molecules, and the $m$th molecule has $K^{(m)}$ observed nuclei of the given type. The true value at the $k$th nucleus of molecule $m$ is $\delta_k^{(m)}$ and the estimated value is $\hat{\delta}_k^{(m)}$

Mean per-molecule Mean Absolute Error:

$$\text{MOL MAE} = \frac{1}{M}\sum_{m=1}^{M}\frac{1}{K^{(m)}}\sum_{k=1}^{K^{(m)}}|\hat{\delta}_k^{(m)} - \delta_k^{(m)}| \tag{1}$$

Mean per-molecule Root Mean Squared error:

$$\text{MOL RMSE} = \frac{1}{M}\sum_{m=1}^{M}\sqrt{\frac{1}{K^{(m)}}\sum_{k=1}^{K^{(m)}}(\hat{\delta}_k^{(m)} - \delta_k^{(m)})^2} \tag{2}$$

Mean absolute error:

$$MAE = \Big(\sum_{m=1}^{M} K^{(m)}\Big)^{-1}\Bigg(\frac{1}{M}\sum_{m=1}^{M}\sum_{k=1}^{K}|\hat{\delta}_k^{(m)} - \delta_k^{(m)}|\Bigg) \tag{3}$$

# 2 Other methods

## 2.1 HOSE codes

Hierarchically Ordered Spherical Environment (HOSE) codes [1] encode the environment of an atom in a molecule in a sphere-wise manner. Spheres are defined by bond distance, so the atoms one bond away form the first sphere, those two bonds away the second sphere etc. For each atom, information including element symbol and bond order is recorded. These codes are then written in a single character sequence, starting with the center atom and going up as many spheres as requested. A typical HOSE code would be:

```
C-3;=OCN(,*C*N,CC/*NN,*CC,=O&,HHH/*&,&C,H*&,HHH,),HHH//
```

Here, the central atom, for which the HOSE code is generated, is a carbon atom with three neighbours. The first sphere (i.e. the atom in distance of one bond to the central atom) consists of a double bonded (indicated by "=") oxygen and a single-single bonded carbon and nitrogen atom (single-bonds are default). The next sphere is separated by "(" and inside the sphere, the atom bonded to each atom in the previous sphere are separated by ",". So here, no atom is bonded to

the oxygen, an aromatic (indicated by "*") carbon and nitrogen atom are bonded to the carbon atom, and two carbon atoms are bonded to the nitrogen. The further spheres are described in a similar fashion, with the sphere deliminator changing to "/" and ")".

NMR prediction using HOSE codes is done per atom. For each atom in the molecule to predict we want to find atoms in the reference data set which have an environment as similar as possible to the atom in question. This is done by generating the HOSE codes for all atoms in the reference dataset for a sufficiently high number of spheres (typically six are used). We also generate the HOSE code with the maxiumum number of spheres of the atom to predict a shift for. If an with this HOSE code is found in the reference data set, the shifts assigned to this atom is the prediction. If more than one shift exists, the mean of all shifts is taken as the prediction. If none is found, the number of spheres used is reduced by one and the search done again. As soon as a shift is found, the shift is used and the prediction for this atom is finished.

Clearly the higher the number of spheres used the better the prediction. Therefore, the number of spheres used is a confidence measure, with six meaning a very good prediction and one a very weak one. In theory a zero-sphere prediction, where the element and neighbour count of the central atom are the only information used is the lowest level possible, but this is too unspecific to be of any use. Furthermore, a reference data set not having a one-sphere HOSE code for all possible cases is probably not good for the intended purpose. HOSE codes do not encode stereochmistry.

The use of HOSE codes for chemical shift prediction has been demonstrated e.g. in [4, 2]. It is easily possible to include solvents in the prediction by only using shift values from spectra with a certain solvent. The prediction is deterministic, which makes it possible to exactly trace back how a prediction was composed. This is useful since it makes finding errors in the database possible.

For this paper, we have used the HOSE code prediction of the nmrshiftdb2 code, which in turn uses the Chemistry Developement Kit (CDK) [8] for the generation of the HOSE codes. In nmrshiftdb2 molecules are written to a relational database, with an entry for each atom in a table. This table also includes the HOSE code for the atom. Chemical shifts are listed in a separate table and linked to atoms. We can therefore search in the database for all shifts linked to an atom which has a certain HOSE code. If the fallback to lower spheres is needed, we search for shifts linked to atoms the HOSE code of which start with a certain character sequence. The code of nmrshiftdb2 is available at `https://sourceforge.net/p/nmrshiftdb2/code/HEAD/tree/trunk/nmrshiftdb2/`.

### 2.2 DFT Methods

For ab initio calculations we followed the best practices outlined in [5] and [9]. Briefly we perform molecular conformation search using Macromodel [7] identifying the most probable conformers by energy. Each conformation then undergoes DFT-based geometry optimization at the `B3LYP / 6-31+G(d,p)` level of theory via Gaussian16 vA03 [3]. DFT-based gauge-independent atomic orbitals are used to calculate isotropic shielding values for each conformation at the `mPW1PW91 / 6-311+G(2d,p)` level of theory with a PCM solvent model with chloroform solvent (again with Gaussian16 vA03). Isotropic shielding values are Boltzmann-weighted via the calculated molecular energies from this last phase, and the weighted average shielding values are calculated. Following the procedure in [5], we then convert calculated isotropic shieldings to chemical shift values via a linear fit to the experimentally observed values, with two modifications: we use a linear fit method robust to outliers (RANSAC, from Scikit-Learn [6]) and we fit to all the data, as opposed to separate train-test sets. This results in a potential over-estimate of the accuracy of DFT, but this is fine

for our purposes of comparison. The full inputs and outputs of all experiments are provided in the supplementary data section.

## References


[1] W. Bremser. "Hose — a novel substructure code". In: *Anal Chim Acta* 103.4 (1978), pp. 355–365. ISSN: 0003-2670. DOI: https://doi.org/10.1016/S0003-2670(01)83100-7. URL: http://www.sciencedirect.com/science/article/pii/S0003267001831007.

[2] *C13 NMR Prediction.* URL: http://www.modgraph.co.uk/product_nmr_c13.htm.

[3] M. J. Frisch et al. *Gaussian 16, Revision A.03.* Wallingford CT, 2016.

[4] S. Kuhn et al. "Building blocks for automated elucidation of metabolites: machine learning methods for NMR prediction". In: *BMC Bioinformatics* 9 (Sept. 2008), p. 400.

[5] Michael W. Lodewyk, Matthew R. Siebert, and Dean J. Tantillo. "Computational prediction of 1H and 13C chemical shifts: A useful tool for natural product, mechanistic, and synthetic organic chemistry". In: *Chemical Reviews* 112.3 (2012), pp. 1839–1862. ISSN: 00092665. DOI: 10.1021/cr200106v. arXiv: arXiv:1011.1669v3.

[6] Fabian Pedregosa et al. "Scikit-learn: Machine Learning in Python". In: *Journal of Machine Learning Research* 12 (Jan. 2012), pp. 2825–2830. URL: http://jmlr.csail.mit.edu/papers/volume12/pedregosa11a/pedregosa11a.pdf%20http://arxiv.org/abs/1201.0490.

[7] *Schrödinger Release 2018-4, Macromodel.* New York, 2018.

[8] E. L. Willighagen et al. "The Chemistry Development Kit (CDK) v2.0: atom typing, depiction, molecular formulas, and substructure searching". In: *J Cheminform* 9.1 (June 2017), p. 33.

[9] Patrick H Willoughby, Matthew J Jansma, and Thomas R Hoye. "A guide to small-molecule structure assignment through computation of (1H and 13C) NMR chemical shifts." In: *Nature protocols* 9.3 (2014), pp. 643–60. ISSN: 1750-2799. DOI: 10.1038/nprot.2014.042. URL: http://www.ncbi.nlm.nih.gov/pubmed/24556787.
